# Correlation of antigen-specific immune response with disease severity among COVID-19 patients in Bangladesh

**DOI:** 10.3389/fimmu.2022.929849

**Published:** 2022-09-28

**Authors:** Taufiqur Rahman Bhuiyan, Hasan Al Banna, M. Hasanul Kaisar, Polash Chandra Karmakar, Al Hakim, Afroza Akter, Tasnuva Ahmed, Imam Tauheed, Shaumik Islam, Mohammad Abul Hasnat, Mostafa Aziz Sumon, Asif Rashed, Shuvro Ghosh, John D. Clemens, Sayera Banu, Tahmina Shirin, Daniela Weiskopf, Alessandro Sette, Fahima Chowdhury, Firdausi Qadri

**Affiliations:** ^1^ Infectious Diseases Division, International Centre for Diarrhoeal Disease Research Bangladesh (ICDDRB), Dhaka, Bangladesh; ^2^ Department of Genetic Engineering and Biotechnology, Jagannath University, Dhaka, Bangladesh; ^3^ Department of Cardiology, Department of Oncology, Kurmitola General Hospital, Dhaka, Bangladesh; ^4^ Department of Microbiology, Department of Medicine, Mugda Medical College and Hospital, Dhaka, Bangladesh; ^5^ Department of Epidemiology, University of California Los Angeles (UCLA) Fielding School of Public Health, Los Angeles, CA, United States; ^6^ International Vaccine Institute, Seoul, South Korea; ^7^ Institute of Epidemiology, Disease Control and Research (IEDCR), Dhaka, Bangladesh; ^8^ Center for Infectious Disease and Vaccine Research, La Jolla Institute for Immunology (LJI), La Jolla, CA, United States; ^9^ Department of Medicine, Division of Infectious Diseases and Global Public Health, University of California, San Diego (UCSD), La Jolla, CA, United States

**Keywords:** COVID-19, SARS-CoV-2, Bangladesh, activation induced marker, CD4^+^ T cells, MAIT cells

## Abstract

Coronavirus disease 2019 (COVID-19) is a protean disease causing different degrees of clinical severity including fatality. In addition to humoral immunity, antigen-specific T cells may play a critical role in defining the protective immune response against SARS-CoV-2, the virus that causes this disease. As a part of a longitudinal cohort study in Bangladesh to investigate B and T cell-specific immune responses, we sought to evaluate the activation-induced marker (AIM) and the status of different immune cell subsets during a COVID-19 infection. We analyzed a total of 115 participants, which included participants with asymptomatic, mild, moderate, and severe clinical symptoms. We observed decreased mucosal-associated invariant T (MAIT) cell frequency on the initial days of the COVID-19 infection in symptomatic patients compared to asymptomatic patients. However, natural killer (NK) cells were found to be elevated in symptomatic patients just after the onset of the disease compared to both asymptomatic patients and healthy individuals. Moreover, we found a significant increase of AIM^+^ (both OX40^+^CD137^+^ and OX40^+^CD40L^+^) CD4^+^ T cells in moderate and severe COVID-19 patients in response to SARS-CoV-2 peptides (especially spike peptides) compared to pre-pandemic controls who are unexposed to SARS-CoV-2. Notably, we did not observe any significant difference in the CD8^+^ AIMs (CD137^+^CD69^+^), which indicates the exhaustion of CD8^+^ T cells during a COVID-19 infection. These findings suggest that patients who recovered from moderate and severe COVID-19 were able to mount a strong CD4^+^ T-cell response against shared viral determinants that ultimately induced T cells to mount further immune responses to SARS-CoV-2.

## Introduction

Coronavirus disease 2019 (COVID-19) caused by the novel coronavirus SARS-CoV-2 has created an unprecedented global pandemic, causing over 588 million confirmed cases and over 6.4 million deaths as of 12 August 2022 (1). In spite of the number of available COVID-19 vaccines, the spread of the disease is still not controlled everywhere. This disease shows several stages of severity, different systemic nature from other respiratory diseases, and unpredictable outcomes with possible comorbidities including cardiovascular disease, obesity, diabetes, lung diseases, kidney diseases, and cancer (2). These have made the management of COVID-19 patients challenging. Moreover, most of the vaccine efforts so far focused on generating neutralizing antibodies by using different surface proteins only, including spike proteins. Nonetheless, T-cell epitopes are derived from both structural and surface proteins (3). Particularly in SARS-CoV-2, the antigen hierarchy as T-cell recognition targets is more distributed across the proteome (4). It is also found that SARS-CoV-2 allows cell-to-cell viral transmission by inducing host–cell fusion, which may act as a resistance to antibody neutralization. Therefore, an understanding of antigen-specific T-cell response to SARS-CoV-2 is imperative to obtain a complete understanding of the adaptive immune response due to COVID-19. Furthermore, this knowledge will provide insights into the pathogenesis of SARS-CoV-2 infection, which in turn helps us to provide targeted interventions to protect vulnerable populations. Apart from that, a thorough understanding of T-cell immune response is needed for vaccine design and evaluation of the next generation of broad-spectrum candidate vaccines.

There are a number of assays conventionally used for measuring the quantity and quality of antigen-specific T cells in humans, either at the single cell level by flow cytometry or enzyme-linked immunospot (ELISpot) and intracellular cytokine staining (ICS) assays or at the population level by enzyme-linked immunosorbent assay (ELISA) (5–7). Several research groups have overcome the limitations of antigen-specific T cells assays based on the upregulation of T-cell receptor (TCR)-stimulated surface markers called activation-induced markers (AIMs), which can effectively determine the overall antigen-specific T-cell response (8). Several studies have successfully used the AIM assays to detect virus-specific, vaccine-specific, or tuberculosis-specific CD4^+^ T cells (9–11).

However, a concern has arisen because a decline of antibodies has been observed within the first few months after recovery from SARS-CoV-2 infection (12, 13). Apart from natural infections, the effectiveness of currently available vaccines has also been reported to decline over time. Initial studies had shown that a single dose of vaccines was overall 41% effective at preventing SARS-CoV-2 infections and 95% effective at preventing COVID-19-related death (14). Nonetheless, more recent studies on the duration of effectiveness of vaccines against SARS-CoV-2 infection decreased from 1 to 6 months after full vaccination by 21% (15). The alarming fact is that vaccines are found to respond very differently to different variants of the virus reported by a study that investigated efficacy after primary immunization with two doses of BNT162b2 (Pfizer, BioNTech), ChAdOx1 nCoV-19 (AstraZeneca), or mRNA-1273 (Moderna) vaccine (16). More specifically, their study reported that vaccine effectiveness against the symptomatic disease was higher for the delta variant than for the omicron variant. Consequently, more studies are essential focusing on different aspects of human immune response to design better vaccines with broad efficacy. We believe that identifying the particular immune cells that are being activated by SARS-CoV-2 may have a substantial impact on evaluating the immune response to COVID-19 and adopting different strategies to design future vaccines. Therefore, to understand the attributes of adaptive immunity, in the present study, we have examined the T-cell responses to activation-induced markers and measured the frequency of different immune cells in the blood and the stability of immune memory in patients suffering from COVID-19 with varying degrees of severity.

## Methods and materials

### Participants and study design

The demographic characteristics (age, sex, and blood group) of the participants are shown in **Supplementary Table 1**. A total of 86 patients (10 of whom expired), 19 healthy controls, and 10 unexposed controls were included in this study. The unexposed controls, also referred to as pre-pandemic controls, were healthy individuals who were enrolled at icddr,b, Dhaka, in prior studies and whose samples were collected before SARS-CoV-2 had been detected in China. The healthy controls were individuals who had no history of COVID-19 during the pandemic, exhibited no clinical symptoms for at least 2 weeks before enrollment, and further tested negative for SARS-CoV-2 by reverse transcription polymerase chain reaction (RT-PCR) during enrollment. The COVID-19 patients were individuals whose nasopharyngeal swabs tested positive for SARS-CoV-2 by RT-PCR. The enrolled patients were further classified using the WHO guidelines into four categories, i.e., asymptomatic, mild, moderate, and severe, depending on the clinical symptoms and oxygen saturation during enrollment (17). The patients were enrolled from Mugda Medical College and Hospital, Dhaka, Bangladesh, and Kurmitola General Hospital, Dhaka, Bangladesh, while the non-hospitalized patients were enrolled from the community.

Blood samples were collected, and peripheral blood mononuclear cells (PBMCs) were isolated and cryopreserved from the patients on Days 1 (day of enrollment), 7, 14, and 28 and from healthy controls on Day 1. The frequency of different T-cell subsets was analyzed from freshly isolated PBMCs of all healthy controls and patients at all day points. Depending on the availability of PBMCs, the frequency of natural killer (NK) cells (n = 40) and mucosal-associated invariant T (MAIT) cells (n = 33) were also analyzed. In addition, SARS-CoV-2 specific T-cell responses were also evaluated using AIM assay in a subset (every fourth/fifth) of COVID-19 patients from each category, i.e., asymptomatic (n = 5, every fifth), mild (n = 6, every fourth), moderate (n = 6, every fourth), and severe (n = 15, alternate). Samples from all the expired patients (n = 10), unexposed controls (n = 10), and healthy controls (n = 10) were also assessed using an AIM assay in order to compare the responses with those from patients.

This study was approved by the Institutional Review Board (IRB) of icddr,b and the Directorate General of Health Services (DGHS), Bangladesh. All procedures were performed after written consent had been obtained from the study participants before enrollment.

### Isolation of peripheral blood mononuclear cells

Blood samples were collected in a sodium-EDTA tube, and isolation of PBMCs was performed by density gradient method using Ficoll-Paque PLUS (Cytiva, Marlborough, MA, USA). Before cells were isolated, plasma was separated using centrifugation. After plasma was separated, the whole blood was diluted at a 1:1 ratio using R-10 media (89% Roswell Park Memorial Institute (RPMI) 1640 (Gibco, Grand Island, NY, USA), 10% fetal bovine serum (FBS; Gibco), and 1% penicillin–streptomycin (Gibco)). Isolated PBMCs were resuspended to a concentration of 1 × 10^6^ cells/ml in RPMI complete medium (87% RPMI 1640, 10% FBS, 1% penicillin–streptomycin, 1% sodium pyruvate (Gibco), and 1% l-glutamine (Gibco)). Fractions of the cells were used fresh for flow cytometry, and the rest of the cells were cryopreserved using the cryoprotectant (90% FBS and 10% dimethyl sulfoxide (DMSO)). These cryopreserved cells were later used in different immune assays, including the AIM assay.

### Flow cytometry and T-cell phenotyping

All immunophenotyping experiments were performed on FACSAria Fusion instruments (BD Biosciences, San Jose, CA, USA). Freshly separated PBMCs were stained with fluorochrome-tagged antibodies, which are listed in **Supplementary Table 2**. After staining, the cells were washed with PBS with 2% FBS (fluorescence-activated cell sorting (FACS) buffer), and antibody-tagged cells were fixed using Cytofix (BD Biosciences, Cat# 554655). Then the data were acquired using the FACSDiva software program the next day. During analyses, live singlet lymphocytes were gated to quantitate different types of T cells (see representative gating in **Supplementary Figure 1**).

Helper T cells were gated as CD19^−ve^CD3^+ve^CD4^+ve^ cells, and cytotoxic T cells were gated as CD19^−ve^CD3^+ve^CD8^+ve^. Follicular helper T cells are CXCR5^+ve^ cells gated on helper T cells. Fluorochrome-tagged antibodies that were used to stain MAIT cells are listed in **Supplementary Table 3**. MAIT cells were gated on live singlets as CD3^+ve^CD8^+ve^ TCR Vα7.2^+ve^CD161^+ve^ (see representative gating in **Supplementary Figure 2**). Again, antibodies used for NK cell staining are listed in **Supplementary Table 4**. During NK cell analysis, CD19 and CD14 were dumped using a single channel, and then the NK cells were gated as CD3^−ve^CD16^+ve^CD56^+ve^. Lastly, the central memory T (Tcm) and effector memory T (Tem) cells were gated as CD45RO^+ve^CD27^+ve^ and CD45RO^+ve^CD27^−ve^, respectively, on either CD4 or CD8 cells. All the phenotyping results are expressed as a percentage of the parent population. The acquired data were analyzed with FlowJo software (version 10.6.1, TreeStar Inc., Ashland, OR, USA).

### Activation-induced marker assay

To identify and quantitate the SARS-CoV-2-specific CD4^+^ as well as CD8^+^ T-cell responses, TCR-dependent AIM assays (4) were performed using cryopreserved PBMCs. The PBMCs of COVID-19 patients and other control samples were stimulated with four different SARS-CoV-2-specific peptide megapools (MPs). SARS-CoV-2 specific CD4 peptide MPs were divided into two groups: spike protein as spike MP and all other polyproteins as non-spike MPs. Again, the antigen-specific analyses of CD8 T cells were performed using the class I peptide megapool prepared from the whole virus proteome and consisting of 628 peptides. The megapool was split into two parts consisting of 314 peptides each, CD8-A MP and CD8-B MP (18). Cryopreserved cells were thawed and rested overnight in a 37°C incubator supplemented with 5% CO_2_. The next day, they were distributed into a 96-well U-bottom plate in a total of 0.3 × 10^6^ PBMCs per well, and different peptide MPs were added at 1 mg/ml concentration. An equimolar quantity of DMSO (vehicle) and phytohemagglutinin (PHA) were used as negative and positive controls, respectively. Moreover, cytomegalovirus-derived peptides, like cytomegalovirus (CMV) CD4 MP and CMV CD8 MP, were used as positive controls to compare the stimulations with the SARS-CoV-2 MPs. The PBMCs were stimulated for 24 h in a 37°C incubator supplemented with 5% CO_2_.

The stimulated cells were stained for 1 h at 4°C in the dark with a cocktail of antibody panels (**Supplementary Table 5**) targeting the AIM^+^ T cells. Following the surface staining, cells were washed with PBS with 2% FBS and fixed using Cytofix (BD Biosciences). Later, data acquisition was carried out in a FACSAria Fusion cytometer through FACSDiva software. Data analyses were performed using FlowJo 10.6.1. During analysis, live singlet lymphocytes were gated, and CD14^+ve^ and CD16^+ve^ cells were discarded by a dump channel. Then both OX40^+ve^CD40L^+ve^ and OX40^+ve^CD137^+ve^ cells were gated on CD4^+ve^ cells, while CD137^+ve^CD69^+ve^ cells were gated on CD8^+ve^ cells (see representative gating in **Supplementary Figure 3**). We measured the stimulation index (SI) for each participant for every type of stimulation to quantify the extent of stimulation. SI has been defined by dividing the percentage of megapool-stimulated AIM^+^ cells by the percentage of DMSO-stimulated AIM^+^ cells. Stimulation due to SARS-CoV-2 CD8 MP was determined using the combined SI data of CD8-A and CD8-B MP.

### Statistical analysis

All statistical analysis was performed using GraphPad Prism 6.0. Flow cytometry figures were generated using FlowJo software and other plots were generated using GraphPad Prism. The statistical tests performed for different experiments are included in the corresponding figures. To compare DMSO versus spike, non-spike, CMV, or PHA in both Unexposed and COVID-19 cases we used Wilcoxon matched-pairs signed rank test because the data were paired and nonparametric. To compare unexposed versus COVID-19 cases we performed a two-tail Mann-Whitney test because the data were unpaired and nonparametric. For comparing between groups, we used ordinary one-way ANOVA with Tukey’s multiple comparison test. We also performed a Mann-Whitney test while comparing immune cell frequency in symptomatic and asymptomatic patients. Again, while comparing different day points of the same category of disease condition we utilized paired t-test as the data was parametric. Moreover, we conducted a one-way ANOVA to compare the immune cell frequency of multiple groups with a single group (healthy control) followed by Dunnett's Multiple Comparison Tests to see the differences compared to control. All tests are specified in the corresponding figure legends. All the statistical analyses were done considering a 95% confidence level.

## Results

### Frequency of helper, cytotoxic, and follicular helper T cells with disease severity and time points

**Figure 1:**
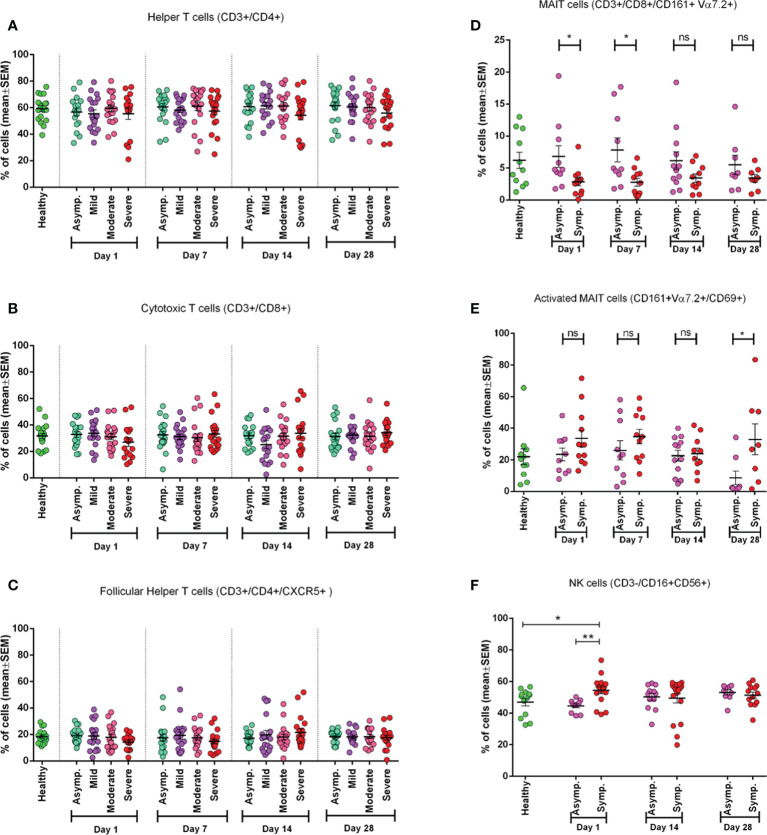
Frequency of different immune cell types in different categories of COVID-19 patients (asymptomatic (n=18), mild (n=19), moderate (n=19), and severe (n=16)) at different day points from the onset of infection and compared with the Healthy controls (n=19). **(A)** Percentage of Helper T cells in CD3+ cells, **(B)** percentage of Cytotoxic T cells in CD3+ cells, **(C)** percentage of Follicular Helper T cells in CD4+ cells. In plots **(D-E)** symptomatic and asymptomatic COVID-19 patients are compared for **(D)** MAIT cells as a percentage of CD8+ cells, **(E)** CD69+ activated MAIT cells (healthy (n=11), asymptomatic (n=10), symptomatic (n=12) patients). **(F)** Percentage of Natural Killer (NK) cells (healthy (n=13), asymptomatic (n=11), symptomatic (n=16) patients). Bars represent the mean value with the Standard Error of Mean. Statistical comparisons were done **(D-F)** using a two-tail Mann-Whitney test. *p <0.05; **p <0.01; ns, non-significant.

We conducted phenotypic analyses of fresh PBMCs for different subsets of T cells. We did not observe any statistically significant difference in frequency for helper, cytotoxic, and follicular T cells among different disease categories in different day points from disease onset (**Figures 1A-C**). It was observed that during the initial days of infection (Day 1), CD8^+^ T cells had a greater reduction in frequencies than CD4^+^ T cells, which happened for neither less severe patients nor in later day points since the infection. This trend was also found for follicular helper T (Tfh) cells (**Figure 1C**) in severe patients where there was decreased frequency of Tfh cells up to the second follow-up (Day 7).

### Mucosal-associated invariant T cell frequency in symptomatic patients during the initial days of infection

We found that MAIT cell frequency tends to decrease significantly (p = 0.0111) in the initial days of infection (both on Day 1 and Day 7). In later days (Day 14 and Day 28), this difference became statistically insignificant in symptomatic patients compared to both asymptomatic patients and healthy participants (**Figure 1D**). We further investigated MAIT cells by including an activation marker (CD69) and observed a higher expression (p < 0.05) of the marker in symptomatic patients throughout the infection compared to asymptomatic patients until 1 month (Day 28) (**Figure 1E**).

### Natural killer cells in symptomatic patients

During the early days of infection, we observed a significantly higher frequency of NK cells in symptomatic patients (p = 0.0014) compared to asymptomatic patients. We also found that NK cell activity was significantly higher in symptomatic patients compared to healthy controls, as expected. However, this difference did not remain significant in the later day points of infection (Days 14 and 28) (**Figure 1F**).

### Frequency of CD4^+^ and CD8^+^ central and effector memory T cells

**Figure 2:**
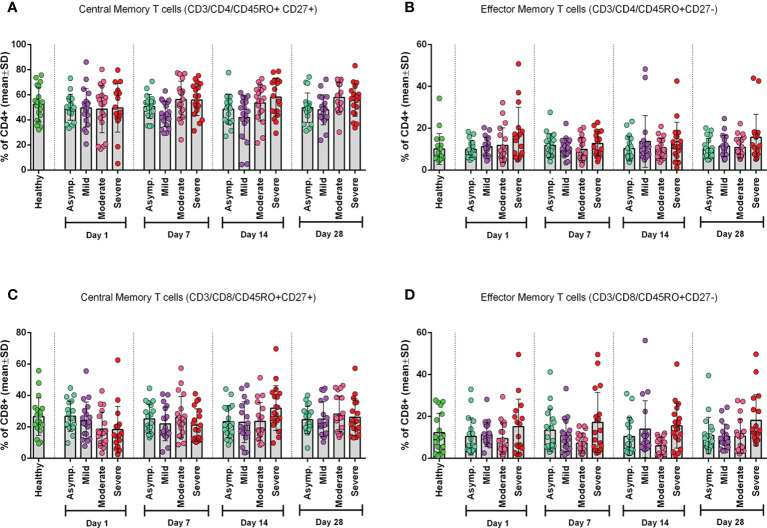
Frequency of Memory T cell subtypes in different categories of COVID-19 patients (asymptomatic (n=18), mild (n=19), moderate (n=19), and severe (n=18)) at different day points from the onset of infection and compared with the Healthy controls. **(A)** CD4+ Central Memory T cells, **(B)** CD4+ Effector Memory T cells, **(C)** CD8+ Central Memory T cells, and **(D)** CD8+ Effector Memory T cells. One-way ANOVA was performed for each of the plots to compare differences of each column with corresponding Healthy control data.

To see the status of the Tcm cells and Tem cells, we measured their frequency in both CD4^+^ and CD8^+^ T cells (**Figure 2**). We found that the frequency of CD4^+^ Tcm cells significantly (p = 0.0119) differed from that of the control group, but CD4^+^ Tem cells did not (**Figures 2A, B**). However, we observed a statistically significant (p = 0.0055) difference for CD8^+^ Tem cells and not for CD8^+^ Tcm cells, when compared with the healthy control group (**Figures 2C, D**).

### Cell recovery tends to decrease after thawing for severe patients

While we thawed cryopreserved PBMCs for AIM assays, we observed a very low count of PBMCs on Day 1 in the case of severe COVID-19 patients compared to healthy patients and other categories. This trend remained the same after overnight resting at 37°C in the CO_2_ incubator. Therefore, we plotted the values and found a significant decrease in recovery of the PBMCs in severe patients compared to healthy controls (**Supplementary Figure 4A**). Recovery after thawing from Day 1 PBMCs of unexposed, healthy, asymptomatic, and dead participants showed no significant differences. This scenario led us to analyze the number of PBMCs per ml of the blood of these samples before cryopreservation, and we again found significantly less number of PBMCs from Day 1 samples of severe cases compared to healthy controls (**Supplementary Figure 4C**). PBMCs on Day 28 from the different patient groups did not show any remarkable difference in the case of the percentages of recovery and number of PBMCs per ml of blood (**Supplementary Figures 4B, D**).

### SARS-CoV-2-specific CD4^+^ T-cell responses in severe COVID-19 patients

**Figure 3:**
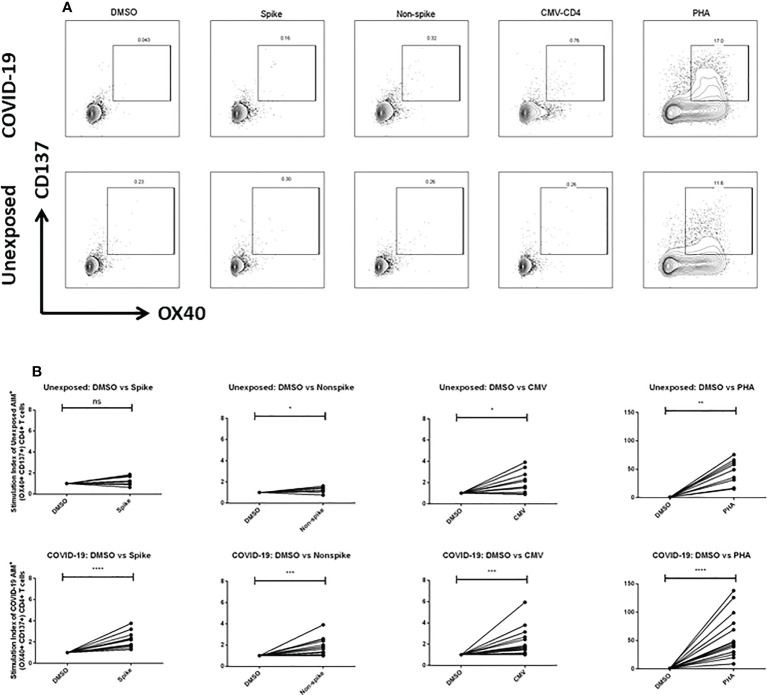
**(A)** Representative plot for Fluorescence-activated cell sorting (FACS) gating for AIM+ (OX40+ CD137+) cells gated on CD4+ T cell; **(B)** AIM+ CD4+ T cell reactivity in unexposed control (n=10) and COVID-19 cases (n=15) between the negative control (DMSO) and different antigen-specific stimulations (Spike, Non-spike MP, CMV, PHA). Wilcoxon matched pairs signed rank test was performed to compare between groups. *p <0.05; **p <0.01; ***p < 0.001; ****p < 0.0001; ns: non-significant.

**Figure 4:**
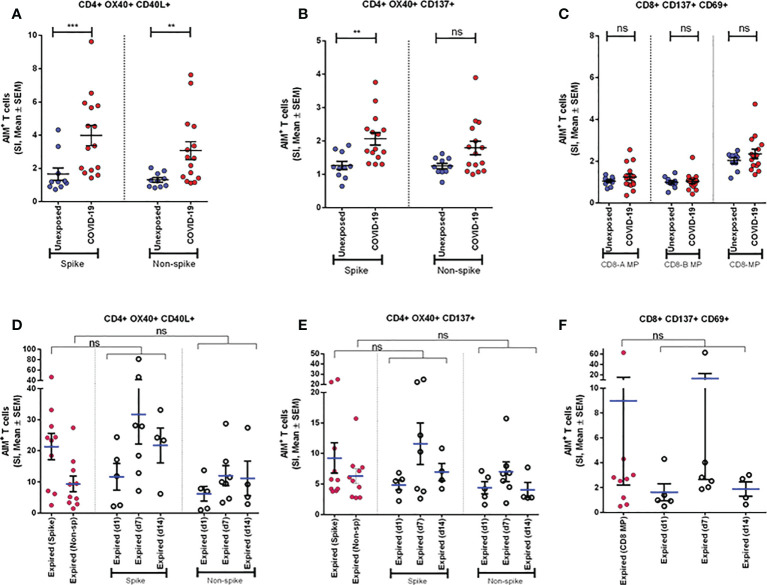
Antigen specific response to different CD4+ and CD8+ AIM markers. **(A-C)** AIM expression in Unexposed (n=10) & COVID-19 participants (n=15). Stimulation Index (SI) quantitation of the AIM+ T cells after stimulation with CD4-nCOV-Spike, Non-spike (CD4-nCOV-all MP), and the class I CD8 peptide MPs (CD8-A and CD8-B). COVID-19 patient samples are collected after one month (day 28) after infection. **(D-F)** AIM expression in “Expired” participants. The left column (purple) shows SI of expired participants (n=10) from samples collected immediate visit before their death. Ordinary one-way ANOVA (with Tukey’s multiple comparison test) was done to compare the groups (for **D-F**). The figure shows the mean SI with error bars representing standard errors of the means (mean ± SEM). Statistical comparisons were performed by a two-tail Mann-Whitney test (for **A-C**). **p<0.01; ***p<0.001; ns: non-significant.

To explore the T cells primed for anti-viral immune response, we evaluated the SARS-CoV-2 specific CD4^+^ T cells in the TCR-dependent AIM assay in COVID-19 patients of different disease severity. We found that AIM^+^ (OX40^+^CD137^+^) CD4^+^ T cells showed a significant increase over the DMSO control in response to both peptide MP spanning the spike domain (spike) and the MP covering the remainder of the SARS-CoV-2 genome (non-spike) (**Figures 3A, B**). When compared with the unexposed response to OX40^+^CD137^+^ of recovered severe patients (Day 28), we found statistically significant differences for spike (p = 0.002), but not for non-spike MPs (**Figure 4B**). Again, both SARS-CoV-2 spike and non-spike reactive AIM^+^ (OX40^+^CD40L^+^) CD4^+^ T cells also showed a significant increase over DMSO control (**Supplementary Figure 5**). Similarly, CMV MP and PHA were also used as positive controls. Notably, SI was also found to be significantly higher for both spike (p = 0.0007) and non-spike (p = 0.0054) MPs in response to this (OX40^+^CD40L^+^) AIM panel in severe COVID-19 patients compared to the unexposed participants (**Figure 4A**). In the case of both AIM panels, the unexposed donors consistently responded to the CMV peptide MP and the PHA superantigen significantly over the DMSO control (**Supplementary Figure 5A, B**). Therefore, according to data, severe COVID-19 patients consistently had a substantial CD4^+^ T-cell response against SARS-CoV-2 after 1 month from the onset of the infection.

### SARS-CoV-2-specific CD8^+^ T-cell responses in severe COVID-19 patients

To measure the SARS-CoV-2 specific CD8^+^ T cells in unexposed and severe COVID-19 patients, we used CD8-A and CD8-B peptide MPs where the whole virus proteome was split between these groups of MPs. Though both unexposed and COVID-19 patients responded consistently to CMV and PHA, they did not respond to CD8-A and CD8-B MPs substantially (**Supplementary Figure 6A, B**). When we combined the SI for CD8-A and CD8-B MPs, we obtained a somewhat higher AIM^+^ (CD137^+^CD69^+^) CD8^+^ T-cell response, but it was not statistically significant (**Figure 4C**). In essence, when comparing the recovered severe COVID-19 patients to the unexposed participants, antigen-specific T-cell studies revealed a predominant role of CD4^+^ T cells over CD8^+^ T cells.

### SARS-CoV-2-specific CD4^+^ and CD8^+^ T-cell response in patients who had expired

We compared the AIM assay in COVID-19 patients who expired on different days after the onset of symptoms. Very high levels of CD4^+^ AIMs (OX40^+^137^+^ and OX40^+^CD40L^+^) were observed on Day 7 and Day 14 after the onset of symptoms to spike and non-spike proteins. Similarly, we observed that CD8^+^ AIMs (CD137^+^CD69^+^) were also upregulated on Day 7 after the onset of symptoms (**Figures 4D-F**). In addition, we used one-way ANOVA to see the differences in response to spike and non-spike MPs among respective groups of day points. The mean of SI given by samples taken on the last visit before their death for all 10 expired patients showed no significant differences with the responses found in individual day points. This result indicated that deceased patients generally had a very high antigen-specific T-cell response before death and looked similar when compared in combination as well as individually at different day points since infection.

### AIM^+^ T-cell response to spike generated over time

**Figure 5:**
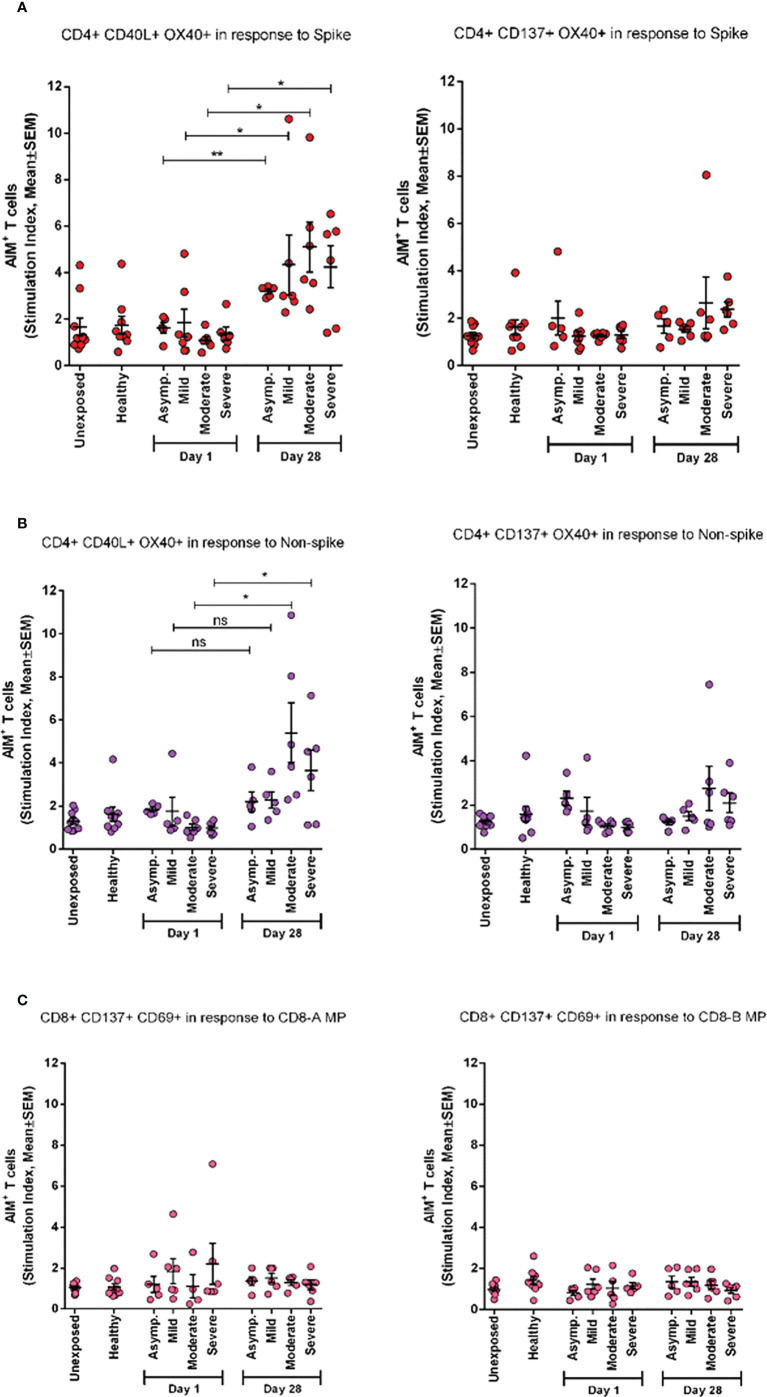
AIM expression in different categories of COVID-19 patients (asymptomatic (n=5), mild (n=5), moderate (n=6), and Severe (n=6)) immediately after infection (Day1) and after one month of infection (Day 28) and compared with both unexposed (n=10) and healthy (n=9) controls. **(A)** Stimulation of AIM+ (OX40+ CD40L+ and OX40+ CD137+) CD4+ T cells in response to Spike antigen; **(B)** Stimulation of AIM+ (OX40+ CD40L+ and OX40+ CD137+) CD4+ T cells in response to non-spike megapool peptides; **(C)** Stimulation of AIM+ (CD137+ CD69+) CD8+ T cells in response to CD8-A and CD8-B megapool peptides. Paired t test is done to compare statistically groups. *p<0.05; **p<0.01; ns: non-significant.

To investigate the periodic scenario of the COVID-19 disease condition from every category, we performed the AIM assay for all categories as well as for healthy controls in the presence of both spike and non-spike MPs. When we compared the SI against spike MPs by paired analysis between Day 1 and Day 28 of infection, we found that every category responded significantly for AIM^+^ (OX40^+^CD40L^+^) CD4^+^ T cells (**Figure 5**). This conveys the message that the T-cell response was augmented due to the COVID-19 infection over time. Though the AIM^+^ T-cell response looked somewhat elevated in every case for Day 28, it was not statistically significant for either non-spike MPs or another AIM panel (OX40^+^CD137^+^).

### Activation of follicular helper T cells due to COVID-19

**Figure 6:**
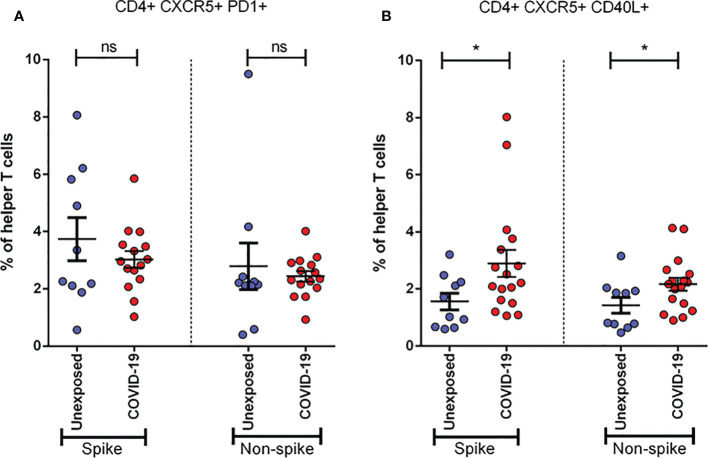
Activation of Follicular Helper T cells (Tfh) in unexposed controls (n= 10)& severe COVID-19 patients (n=15). Samples were collected after one month (Day 28) of infection and response was observed for stimulation by both Spike and Non-spike antigens. **(A)** Frequency of CXCR5+ PD1+ cells in helper T cells; **(B)** Frequency of CXCR5+ CD40L+ cells in helper T cells. Statistical comparisons were done using a two-tail Mann-Whitney test. *p<0.05; ns: non-significant.

To determine the role of Tfh cells in recovered severe COVID-19, we performed the same stimulation experiment utilizing both spike and non-spike MPs and analyzed the cultured cells by flow cytometry using two different activation markers. We found that the frequency of CXCR5^+^CD40L^+^ cells tends to increase significantly (p < 0.05 for both spike and non-spike), while CXCR5^+^PD1^+^ helper T cells tend to decrease, though not significantly, after 1 month of infection (**Figure 6**). These data suggest the possibility of the role played by Tfh cells against SARS-CoV-2.

## Discussion

Understanding of adaptive immunity to COVID-19 has increased but remains limited and unclear, especially in acute and convalescence COVID-19 patients. As the study was exploratory, the antigen-specific antibody and T-cell data suggest the following conditions: i) AIMs may limit COVID-19 severity; ii) with prominent roles, SARS-CoV-2-specific helper T cells are associated with less COVID-19 severity (19); iii) aging and lack of naive T cell number are possibly linked to the unsuccessful synchronized AIM responses that result in increased vulnerability to severe COVID-19. These findings have significant involvement both to understand immunity and pathology to novel coronavirus and in designing an effective COVID-19 vaccine. Moreover, cellular immunity mediated by T cells (20, 21) and memory B cells (22, 23) plays a crucial role in the resolution of SARS-CoV-2 infections. Initially, for the development of vaccines against COVID-19, most of the focus was given to the role of neutralizing antibodies. Nonetheless, evidence suggests that neutralizing capabilities were lost both over time and in response to new variants. All the evidence gathered so far suggests that for durable and broad protection from old infections or by a vaccine, T cells can play a better role (4, 24–26). Considering the continuous generation of new variants of SARS-CoV-2, T cell-based immunity should be the focus of immune surveillance as well as vaccine development. This is because it was found that 70% to 80% of the CD4^+^ and CD8^+^ T cell epitopes in the spike protein were not affected by Omicron mutations, and immune responses generated by T cells were mostly preserved (27). The current study can provide some valuable insight regarding T-cell immunity because among different cellular assays, AIM approaches can provide a more in-depth characterization of antigen-specific T cells and their subtypes.

In our earlier reports, we presented clinical, genomic, and humoral antibody responses to COVID-19 (28–30). This is the first longitudinal study in Bangladesh that has been carried out to evaluate the adaptive T-cell immune responses in patients with differing levels of severity of illness after SARS-CoV-2 infection. Bangladesh is similar to other countries globally regarding age, gender, and comorbid conditions (31, 32). However, the death rate due to COVID-19 is low in Bangladesh, as the demographic characteristics of our population are different compared to those of other countries, and the elderly population (>50 years) is only 10%–15% (33). Therefore, this study is particularly important for a population of similar demographic criteria. Again, when the current study samples were collected in Dhaka city (November 2020 to July 2021), multiple variants of SARS-CoV-2 were prevalent, for example, the Wuhan-like variant (up to February 2021), the B.1.1.7 (Alpha) variant from the UK (during the first half of March 2021), the B.1.351 (Beta) variant from South Africa (from March to May 2021), and the B.1.617 (Delta) variant from India (starting from June 2021) (34). Consequently, the T-cell response data presented in our study do not represent any particular variant of SARS-CoV-2 but rather reflect an overall immunity present in the population. This can be considered another strength of the study. However, we did not confirm this diversity of prevalent variants by genotyping the virus from each participant. Hence, there is a lack of proof behind our assumption and a limitation of the study.

We used two different types of controls, namely, pre-pandemic controls (unexposed to SARS-CoV-2) and healthy controls (collected during the pandemic). The reason behind using these two types of controls was to see any cross-reactive T-cell responses from pre-pandemic cells for SARS-CoV-2, to evaluate SARS-CoV-2 antigen-specific T-cell response, and to compare with COVID-19 patient groups. When we compared the T-cell response of healthy controls with that of the cells preserved from the pre-pandemic time point, there was no significant difference in reactivity to SARS-CoV-2 MPs (**Figure 5**). Thus, it was important to take two types of controls to make our data more credible. Moreover, our data also suggest that either our control groups were equally protected by cross-reactive immunity, as suggested by the literature (24, 35, 36), or both groups were unexposed to seasonal coronaviruses. As we did not evaluate any cross-reactive immunity of our samples, it cannot be confirmed. Furthermore, we could not use pre-pandemic control in the phenotyping analysis of different immune cells, because we had limited amounts of cryopreserved PBMCs. Along with this limitation, the current study has analyzed a small number of samples for the AIM assay in each severity patient group (n = 5/6 for asymptomatic, mild, moderate, and n = 15 for severe) due to high cost, extensive labor, and longer time. Moreover, due to the small sample size in each disease category, it was not possible to conduct any variant-specific AIM assay. Therefore, in the current manuscript, we combined the COVID-19-infected participants (**Figures 1D–F**, **4** and **6**) and reported the data in general, although the outcome might be different if the analyses were performed based on different variants. However, in this study, this was out of the scope and a limitation of the study. A further in-depth study needs to be carried out for understanding variant-specific B- and T-cell immune responses, and we can follow such kinds of analyses in the future. A potential bias may come from flow cytometric data acquisition when control and case samples are stained and run in the cytometer through separate experiments spanning several months. For this study, the AIM assay was performed with case and control samples in a few combined experiments, but phenotyping of fresh PBMCs was performed separately on the day of collection. This had the potential to generate some errors in data; however, we used all kinds of compensation and fluorochrome controls to minimize any unwanted bias among data.

In this study, we investigated the general frequency of T-cell subtypes as well as antigen-specific T-cell responses in different categories of patients based on severity and have compared the responses with those of two different types of control groups—a pre-pandemic control group (referred to as unexposed controls) and healthy control group. One of the most interesting findings from the frequency of T-cell subtype analysis is that MAIT cells were found to decrease in the blood of symptomatic patients (mild, moderate, and severe) compared to asymptomatic patients during the initial days of infection (**Figure 1D**). MAIT cells are innate-like T cells that are supposed to protect during mucosal and viral infections. The reduction of MAIT cell frequency in symptomatic patients suggests that these MAIT cells might provide significant protection during COVID-19 infections. Some other groups have also found similar results including the fact that these changes in MAIT cell frequency positively correlate with the activation of some other innate cells, proinflammatory cytokines, interleukin (IL)-18, with the severity of the disease, and mortality due to COVID-19 (37, 38).

Among other peripheral immune cells, the frequency of NK cells remained quite higher in symptomatic patients compared to both asymptomatic patients and healthy controls subsequently after infection (**Figure 1F**). NK cells are innate effector lymphocytes that can directly target and kill infected cells and can influence adaptive T-cell responses. Thus, our data suggest that NK cells rapidly respond during the acute phase of infections and might also contribute to the immunopathology of symptomatic patients. An initial study by Maucourant et al. characterized NK cells in patients with moderate or severe COVID-19, which supports our findings (39). A more recent study suggested that viral clearance, antibody response, and disease progression correlate with NK cell status in patients with COVID-19 (40). They have also suggested that NK cell dysfunction is linked with increased susceptibility to COVID-19 and plays a key role in the switch from effective to harmful immune responses against SARS-CoV-2. However, our understanding of NK cells in the pathogenesis of COVID-19 remains elusive, which demands further investigation.

This study demonstrates the presence of robust antigen-specific CD4^+^ T-cell responses specific for SARS-CoV-2 in the PBMCs of severe COVID-19 patients. Higher viral loads may develop stronger SARS-CoV-2 specific T-cell responses among patients who had severe disease and may reflect a poor early T-cell response that might be inadequate to clear/control the virus. Consistent with current findings (4), a high frequency of helper T-cell responses specific to spike protein was observed in recovered COVID-19 patients. This is much like influenza infection, where surface hemagglutinin of the virus elicited helper T-cell responses, whereas the greater part of cytotoxic T-cell responses was found specific to viral internal proteins (41). Antigen-specific CD8^+^ T cells recognize and kill host cells already infected by the virus, and according to our results, they became exhausted after 1 month of infection, which corresponds to an earlier study (42). However, the reduction of frequency of CD8^+^ T cells at Day 1 in fresh PBMCs of severe patients, not others, compared to CD4^+^ T cells indicates that the presence of CD8^+^ T cells helps to clear initial viral loads, which also corresponds to a previous study (43). Interestingly, CD4^+^ AIM response between Day 1 and Day 28 in asymptomatic patients was more significant than in all other symptomatic patients (**Figure 5A**). This suggests that a strong T-cell response must have protected them from being sick or developing symptoms.

Understanding the roles, timing, and strength of different subsets of T cells in the protection of SARS-CoV-2 is crucial for the prevention and treatment of a COVID-19 infection. By that time, several vaccines are available and WHO-approved globally, and different countries including Bangladesh are administrating vaccines in the population and trying to increase the coverage rate as much as possible. The current COVID-19 infection is more challenging than prophylaxis. The data presented here suggest that SARS-CoV-2-specific CD4^+^ T cells have an association with resolving acute COVID-19 infection. These findings suggest that the introduction of a vaccine can elicit both helper and cytotoxic T cells specific to SARS-CoV-2, along with protective neutralizing antibodies, and thus may generate immunity to provide an adaptive antiviral immune response in SARS-CoV-2 infection. However, further understanding is required to determine adaptive T-cell immune responses in current vaccines simultaneously and correlate the responses with natural infection. By studying antigen-specific T cells, we can monitor the development of crucial immunological responses. As the helper T-cell responses are significantly diverse, detection of antigen-specific helper T cells with the production of one or more cytokines is likely to remarkably miscalculate the amount of the total antigen-specific response (44).

In the current longitudinal study, we reported that moderate or severe disease patients had significantly higher antibody responses to SARS-CoV-2 RBD compared to mild or asymptomatic infection (28). In further analyses, the kinetics of IgG antibody has shown persistence until Day 270 in moderate and severe patients, and in contrast, mild and asymptomatic participants’ antibodies dropped from Day 180 after the onset of COVID-19 diagnosis. The IgM antibody responses showed transient immune responses and dropped after Day 30 after the onset of diagnosis. Developing IgG and IgM isotypes of antibodies by the patients suggest a key role for CD4^+^ T cells in isotype switching and memory responses after a COVID-19 infection (45). It has been reported earlier that the SARS-CoV-2-specific antibodies along with SARS-CoV-2-specific helper and cytotoxic cells persist for approximately 6–8 months (46). In the current study, we have analyzed adaptive T-cell responses until Day 28 after the onset of diagnosis and showed the COVID antigen-specific T-cell responses, and further time points can be evaluated in our future studies. All 10 individuals who expired in this longitudinal study due to the COVID-19 infection had COVID-19-specific antibody responses prior to death that are comparable to those of other patients, suggesting that their humoral and adaptive immune responses are inadequate to control and clear the infection. As we observed higher responses on Day 7 for AIMs in deceased patients, it may be due to more cytokine production [cytokine storm (CS)], which was creating vulnerable immune responses in these patients. In literature, it has been shown that CS during a COVID-19 infection is triggered by increased production of IL-6, IL-10, TNF-α, IFN-γ, etc. (47). These can be produced by an uncontrolled immune response, like continuous activation and expansion of immune cells, lymphocytes, and macrophages. Although cytokines can be produced by different immune cells like DC, NK, and B cells, along with T cells, CS could be a reason for obtaining high CD4^+^ T-cell activation a few days before the participants’ death. There is evidence that SARS-CoV-2-specific T cells predominantly produced proinflammatory cytokines, like effector and T helper 1 (Th1) cytokines, as well as Th2 and Th17 cytokines (48). We can assume that after CS some days were needed to develop severe systemic inflammation, other complications, and organ failure, which ultimately lead to death.

In summary, we found robust CD4^+^ AIM responses that were observed in COVID-19 patients, and these responses are more in severe patients compared to asymptomatic and mild patients. Patients with moderate and severe disease developed higher levels of AIM responses that may help to generate good humoral responses to clear the infection in COVID-19 patients. Our data also suggest that T-cell response plays a key role as a regulator of disease severity, possibly recovery from SARS-CoV-2 infection, and consequently has the potential to be the new focus for future vaccine design.

## Data availability statement

The original contributions presented in the study are included in the article/**Supplementary Material**. Further inquiries can be directed to the corresponding author.

## Ethics statement

This study was reviewed and approved by Institutional Review Board, International Centre for Diarrhoeal Disease Research, Bangladesh. The patients/participants provided their written informed consent to participate in this study.

## Author contributions

FQ, TB, FC, AA, and TA designed, managed, and supervised the study. AA, TA, IT, AH, MAS, AR, and SG helped to collect the specimens. HB, MK, PK, AH, and SI performed the laboratory work and immunological analyses. TB, HB, MK, and PK analyzed the data. FQ, TB, DW, and TS provided key reagents. TB, HB, and AH drafted the manuscript. FQ, TB, SB, TS, JC, DW, SB, TS, FC, AA, TA, and IT reviewed and planned the manuscript. All authors contributed to the interpretation of results and critical review and revision of the manuscript and have approved the final version.

## Funding

This study was supported by Grant INV-018954 from the Bill and Melinda Gates Foundation, Global Emerging Leader Award (K43TW010362), R01 AI130378 of the National Institutes of Health (NIH), and icddr,b. This work has been supported by NIH contract 75N93019C00065 (A.S and D.W).

## Acknowledgments

The authors would like to thank the Ministry of Health and Family Welfare (MOHFW) of Bangladesh, IEDCR, ideSHi, Kurmitola General Hospital, and Mugda Medical College and Hospital for their continuous support. The authors would also like to thank Daniela Weiskopf for providing the SARS-CoV-2 peptides for carrying out the AIM assay. The authors would also like to express their sincere thanks to the staff members of icddr, b for their dedicated work in the field and laboratory during the pandemic situation. icddr,b is supported by the Governments of Bangladesh, Canada, Sweden, and the UK. The funders had no role in study design, data collection and analysis, decision to publish, or preparation of the manuscript.

## Conflict of interest

AS is a consultant for Gritstone Bio, Flow Pharma, ImmunoScape, Avalia, Moderna, Fortress, Repertoire, erson Lehrman Group, RiverVest, MedaCorp, and Guggenheim. La Jolla Institute has filed for patent protection for various aspects of T cell epitope and vaccine design work.

The authors declare that the research was conducted in the absence of any commercial or financial relationships that could be construed as a potential conflict of interest.

## Publisher’s note

All claims expressed in this article are solely those of the authors and do not necessarily represent those of their affiliated organizations, or those of the publisher, the editors and the reviewers. Any product that may be evaluated in this article, or claim that may be made by its manufacturer, is not guaranteed or endorsed by the publisher.

## Supplementary material

Supplementary Figure 1 Representative plot and gating strategy for T cell phenotyping.

Supplementary Figure 2 Representative plot and gating strategy for Mucosa Associated Invariant T (MAIT) cell.

Supplementary Figure 3 Representative plot and gating strategy for Activation Induced Marker (AIM) assay.

Supplementary Figure 4 Percentages of PBMCs recovery of different disease groups (healthy controls, n = 9; asymptomatic, n = 4; mild, n =7; moderate, n =7; severe, n = 6; expired n = 9) after thawing at day 1 (4A) and day 28 (4B). Number of PBMCs (in million) per mL of blood from the disease groups at day 1 (4C) and day 28 (4D). Dunnett’s Multiple Comparison Tests were performed in both cases to compare between groups and *p < 0.05.

Supplementary Figure 5
**(A)** Presentative plot for Fluorescence-activated cell sorting (FACS) gating for AIM+ (OX40+ CD40L+) cells gated on CD4+ T cell; **(B)** AIM+ CD4+ T cell reactivity in unexposed control (n=10) and COVID-19 cases (n=15) between the negative control (DMSO) and different antigen-specific stimulations (Spike, Non-spike MP, CMV, PHA). Wilcoxon matched-pairs signed rank test was performed to compare between groups. *p < 0.05; **p < 0.01; ***p < 0.001; ns: non-significant.

Supplementary Figure 6
**(A)** Presentative plot for Fluorescence-activated cell sorting (FACS) gating for AIM^+^ (CD69^+^ CD137+) cells gated on CD8^+^ T cell; **(B)** AIM^+^ CD8^+^ T cell reactivity in unexposed control (n = 10) and COVID-19 cases (n = 15) between the negative control (DMSO) and different antigen-specific stimulations (CD8-A MP, CD8-B MP, CMV, PHA). Wilcoxon matched-pairs signed rank test was performed to compare between groups. *p < 0.05; **p < 0.01; ***p < 0.001; ns: non-significant.
