# Supplementary figures and images for: Correlation of antigen-specific immune response with disease severity among COVID-19 patients in Bangladesh
Source: Front Immunol. 2022 Sep 28;13:929849. doi: 10.3389/fimmu.2022.929849 (PMC9554593; doi:10.3389/fimmu.2022.929849)

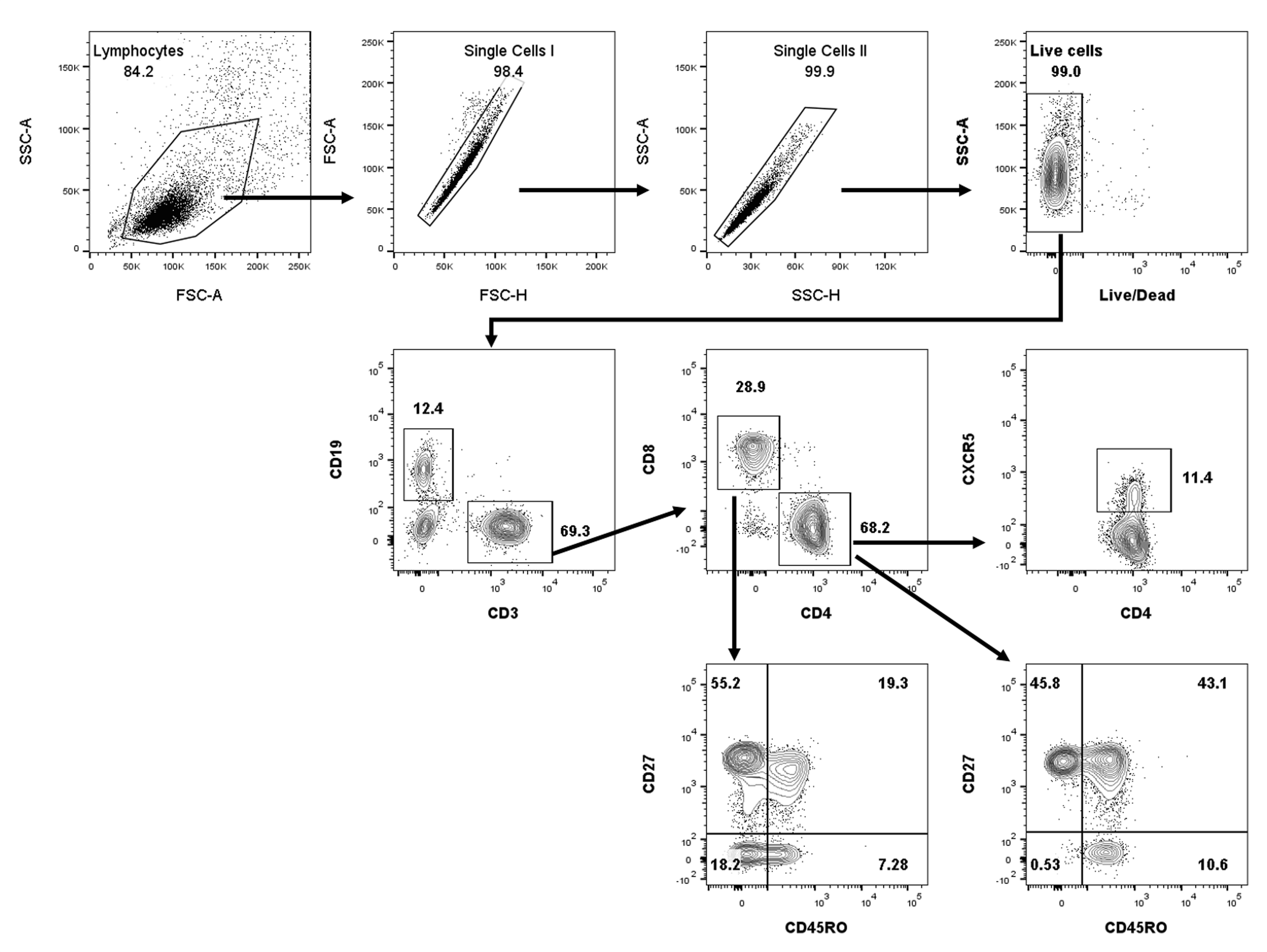

Supplement: Supplementary Figure 1 — Representative plot and gating strategy for T cell phenotyping. [file Image_1.tif]

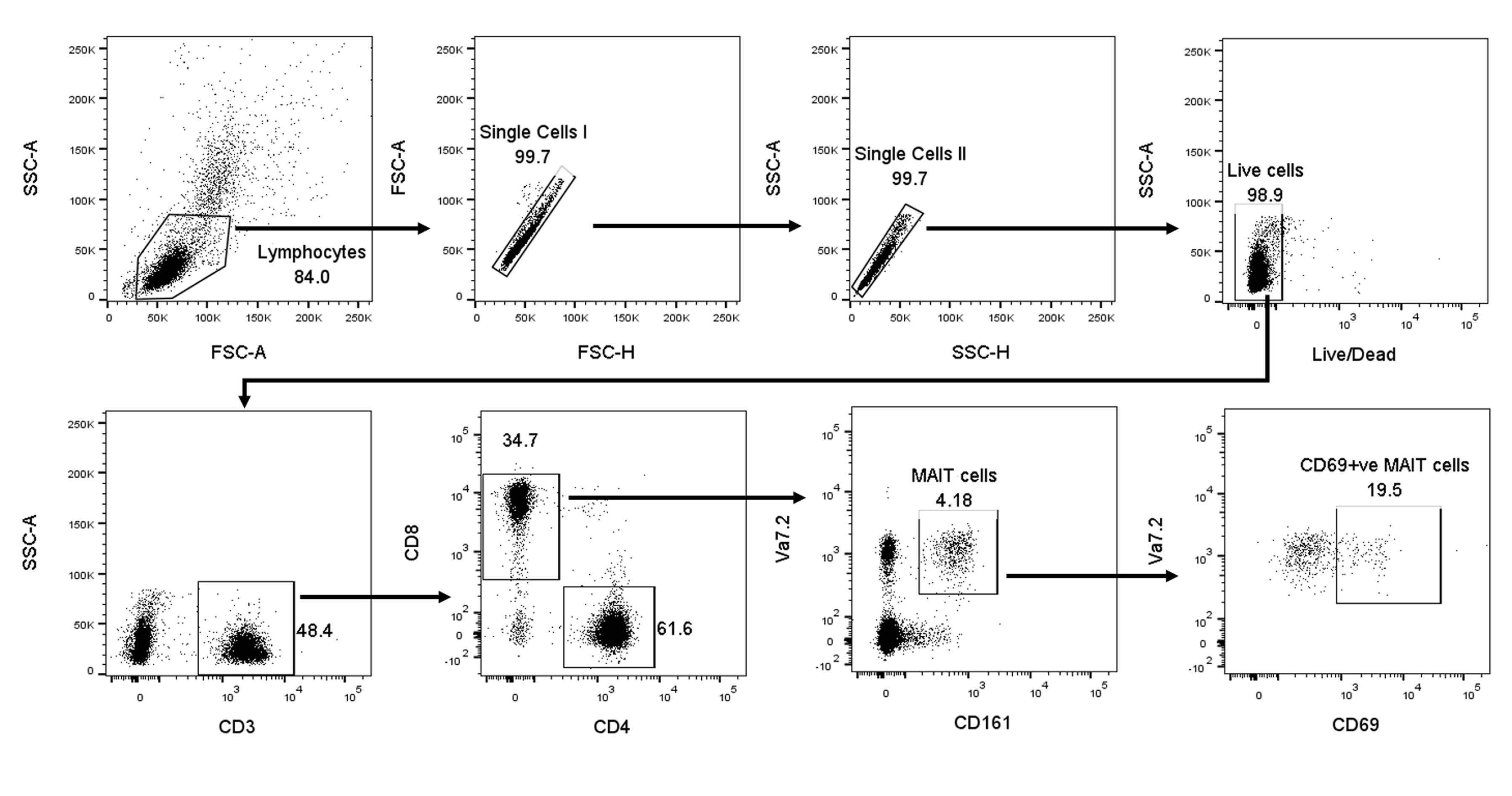

Supplement: Supplementary Figure 2 — Representative plot and gating strategy for Mucosa Associated Invariant T (MAIT) cell. [file Image_2.tif]

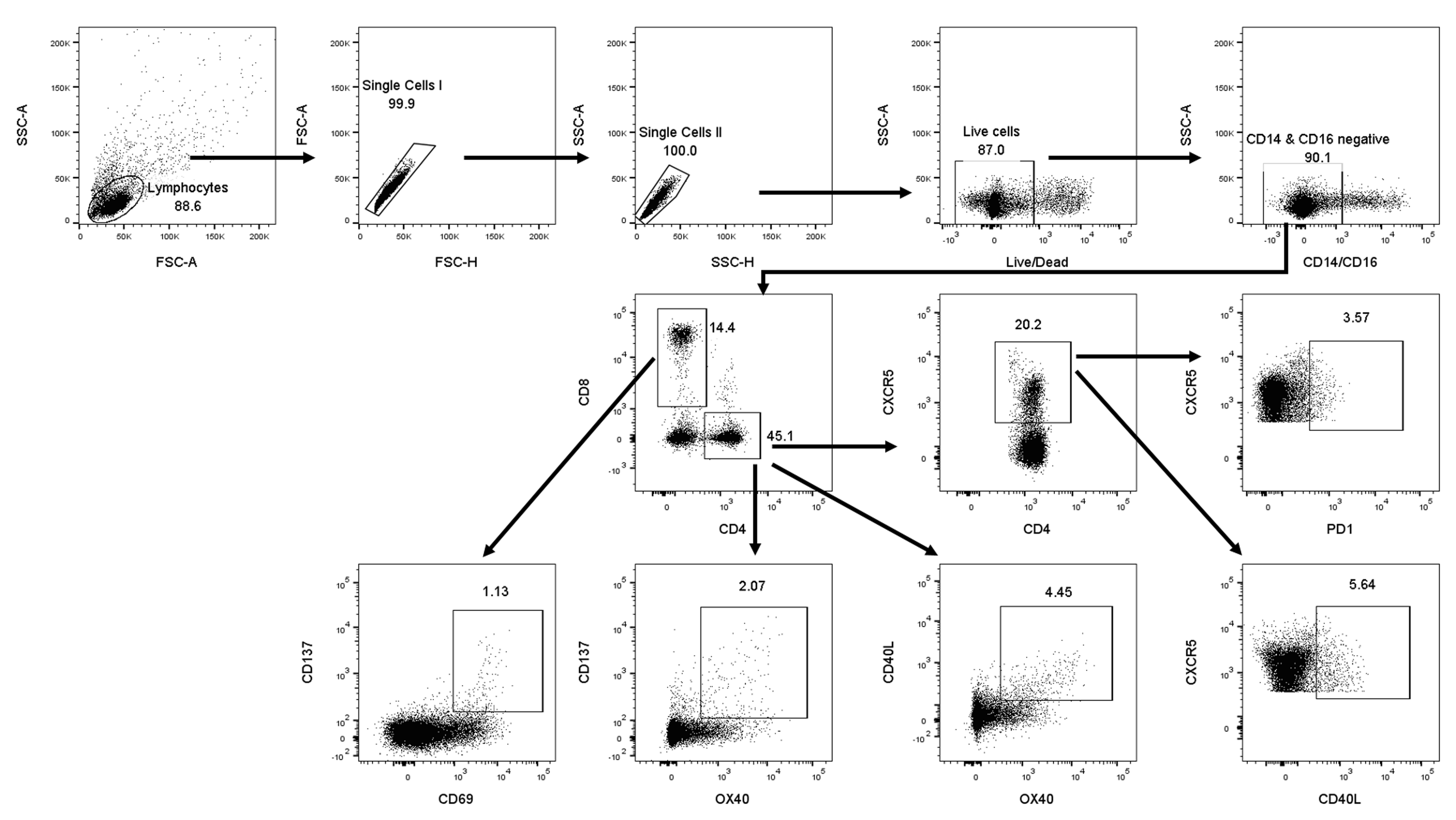

Supplement: Supplementary Figure 3 — Representative plot and gating strategy for Activation Induced Marker (AIM) assay. [file Image_3.tif]

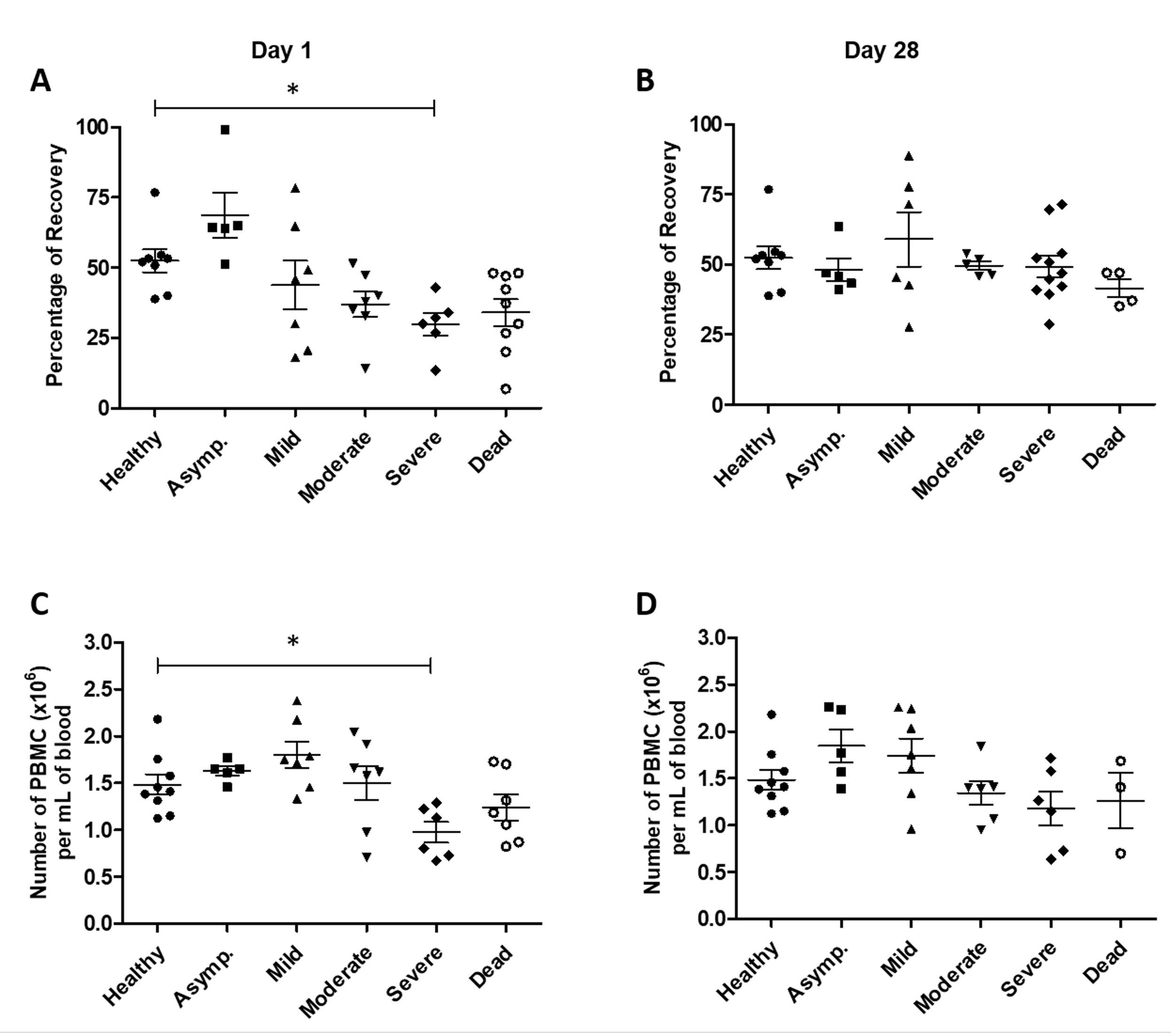

Supplement: Supplementary Figure 4 — Percentages of PBMCs recovery of different disease groups (healthy controls, n = 9; asymptomatic, n = 4; mild, n =7; moderate, n =7; severe, n = 6; expired n = 9) after thawing at day 1 (4A) and day 28 (4B). Number of PBMCs (in million) per mL of blood from the disease groups at day 1 (4C) and day 28 (4D). Dunnett’s Multiple Comparison Tests were performed in both cases to compare between groups and *p < 0.05. [file Image_4.tif]

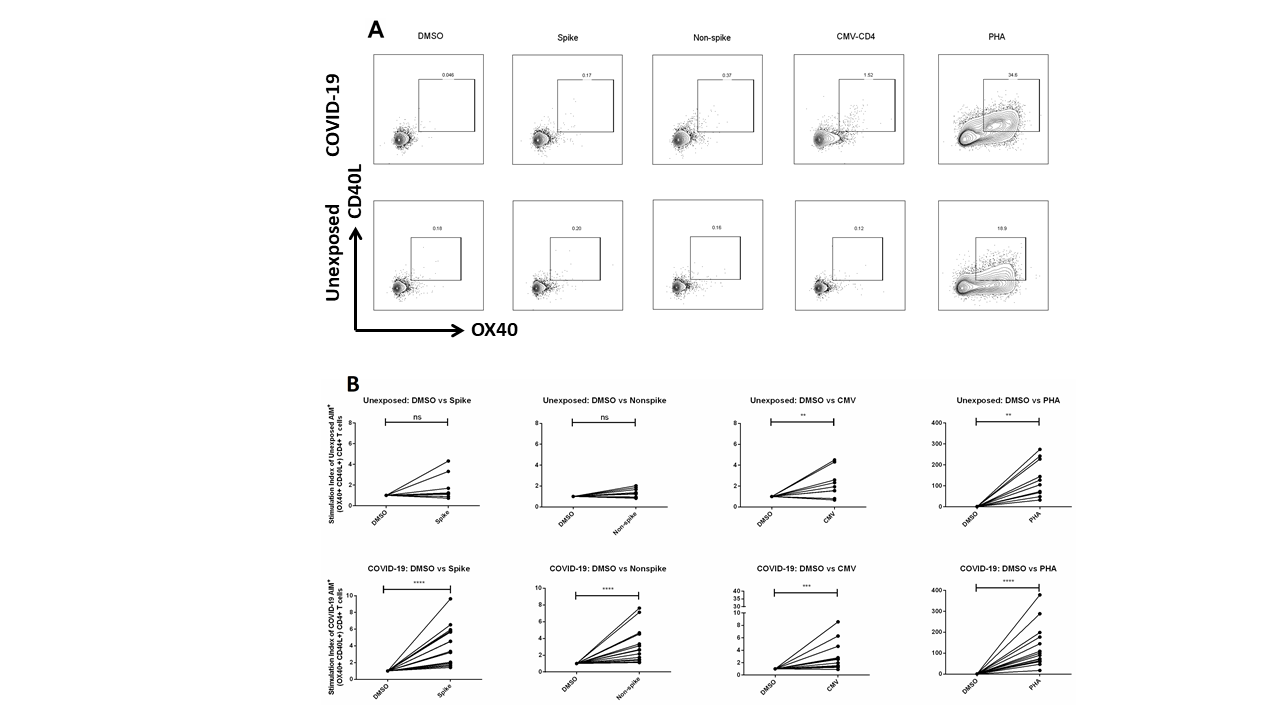

Supplement: Supplementary Figure 5 — (A) Presentative plot for Fluorescence-activated cell sorting (FACS) gating for AIM+ (OX40+ CD40L+) cells gated on CD4+ T cell; (B) AIM+ CD4+ T cell reactivity in unexposed control (n=10) and COVID-19 cases (n=15) between the negative control (DMSO) and different antigen-specific stimulations (Spike, Non-spike MP, CMV, PHA). Wilcoxon matched-pairs signed rank test was performed to compare between groups. *p < 0.05; **p < 0.01; ***p < 0.001; ns: non-significant. [file Image_5.tif]

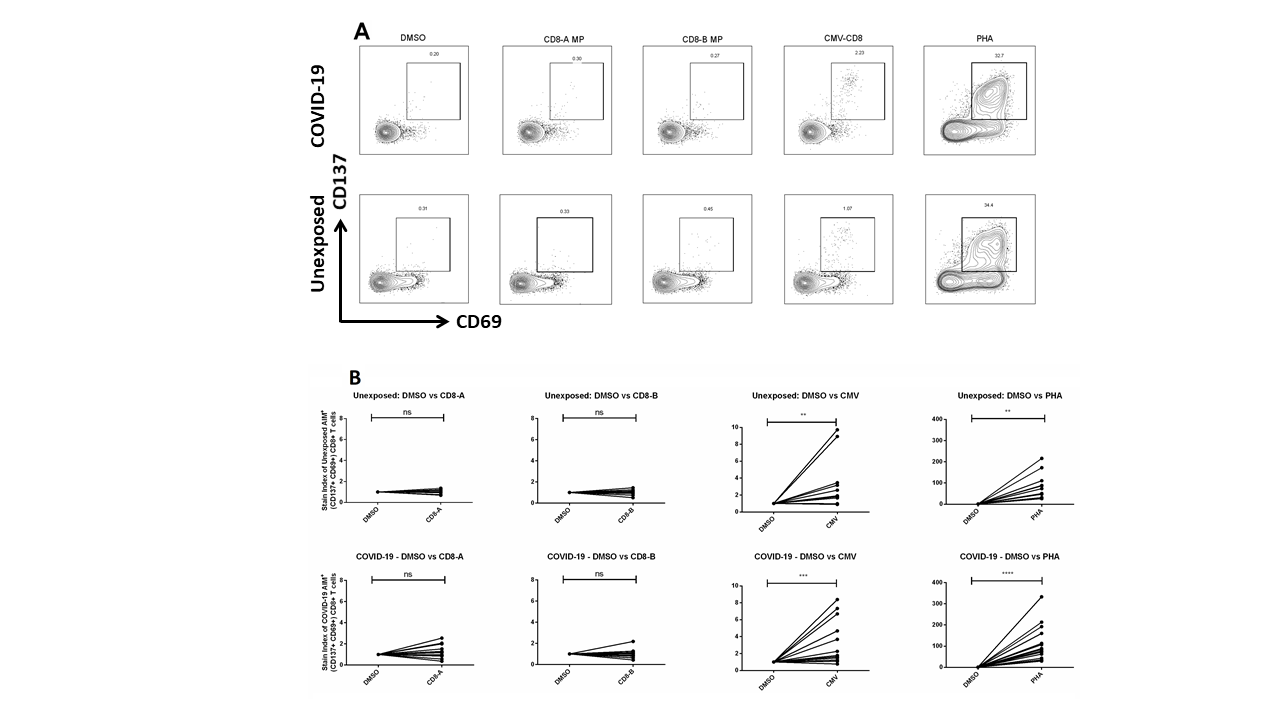

Supplement: Supplementary Figure 6 — (A) Presentative plot for Fluorescence-activated cell sorting (FACS) gating for AIM+ (CD69+ CD137+) cells gated on CD8+ T cell; (B) AIM+ CD8+ T cell reactivity in unexposed control (n = 10) and COVID-19 cases (n = 15) between the negative control (DMSO) and different antigen-specific stimulations (CD8-A MP, CD8-B MP, CMV, PHA). Wilcoxon matched-pairs signed rank test was performed to compare between groups. *p < 0.05; **p < 0.01; ***p < 0.001; ns: non-significant. [file Image_6.tif]
