# Supplementary material for: Correlation of antigen-specific immune response with disease severity among COVID-19 patients in Bangladesh
Source: Front Immunol. 2022 Sep 28;13:929849. doi: 10.3389/fimmu.2022.929849 (PMC9554593; doi:10.3389/fimmu.2022.929849)
Supplement: Supplementary file 7 [file DataSheet_1.docx]

**Supplementary Data:** Bhuiyan et al.

**Title:**

**Correlation of antigen-specific immune response with disease severity among COVID-19 patients in Bangladesh**

Taufiqur Rahman Bhuiyan^1¶^, Hasan Al Banna^1¶^, M Hasanul Kaisar^1^, Polash Chandra Karmakar^1^, Al Hakim^1,2^, Afroza Akter^1^, Tasnuva Ahmed^1^, Imam Tauheed^1^, Shaumik Islam^1^, Mohammad Abul Hasnat^3^, Mostafa Aziz Sumon^3^, Asif Rashed^4^, Shuvro Ghosh^4^, John D Clemens^1,5,6^, Sayera Banu^1^, Tahmina Shirin^7^, Daniela Weiskopf^8^, Alessandro Sette^8,9^, Fahima Chowdhury^1#^, Firdausi Qadri^1,#,*^

^1^International Centre for Diarrhoeal Disease Research Bangladesh (icddr,b), Dhaka, Bangladesh

^2^ Department of Genetic Engineering and Biotechnology, Jagannath University, Dhaka, Bangladesh

^3^Kurmitola General Hospital, Dhaka, Bangladesh

^4^Mugda Medical College & Hospital, Dhaka, Bangladesh

^5^UCLA Fielding School of Public Health, Los Angeles, CA, USA

^6^International Vaccine Institute, Seoul, South Korea

^7^Institute of Epidemiology, Disease Control and Research (IEDCR), Dhaka, Bangladesh

^8^Center for Infectious Disease and Vaccine Research, La Jolla Institute for Immunology (LJI), La Jolla, CA 92037, USA

^9^Department of Medicine, Division of Infectious Diseases and Global Public Health, University of California, San Diego (UCSD), La Jolla, CA 92037, USA

**Running title:**  Antigen-specific immune response due to COVID-19

^¶^Co-first author; ^#^Senior author; ^*^Corresponding author

^*^Dr. Firdausi Qadri, PhD

Senior Scientist and Head, Mucosal Immunology and Vaccinology Unit,

Infectious Diseases Division

International Centre for Diarrhoeal Disease Research, Bangladesh (icddr,b),

68, Shaheed Tajuddin Ahmed Sarani, Mohakhali, Dhaka 1212, Bangladesh.

Tel: +880 (0)2-2222-77001-10, Ext 2431; Email: [fqadri@icddrb.org](mailto:fqadri@icddrb.org)

**Supplementary Table 01: Demographic information of the participants of this study**

| **Variables** | | **COVID-19 patients**  **(n=86)** | | | | | **Healthy Control**  **(n=19)** | **Unexposed (Pre-pandemic)**  **(n=10)** |
| --- | --- | --- | --- | --- | --- | --- | --- | --- |
|  |  | **Asymptomatic**  **(n=19)** | **Mild**  **(n=19)** | **Moderate**  **(n=19)** | **Severe**  **(n=19)** | **Expired**  **(n=10)** |  |  |
| Mean Age (Years) | 45.9 (n=115) | 37 | 45.6 | 49.1 | 55.9 | 58.7 | 42.2 | 31.9 |
| Sex | Male (n=70) | 7 | 12 | 14 | 14 | 6 | 12 | 5 |
|  | Female (n=45) | 12 | 7 | 5 | 5 | 4 | 7 | 5 |
| Blood Group | O+ (n=41) | 5 | 6 | 6 | 7 | 2 | 10 | 5 |
|  | A+ (n=28) | 7 | 5 | 4 | 5 | 3 | 3 | 1 |
|  | B+ (n=37) | 5 | 8 | 8 | 6 | 4 | 5 | 1 |
|  | AB+ (n=6) | 1 | 0 | 1 | 1 | 1 | 0 | 2 |
|  | O- (n=1) | 0 | 0 | 0 | 0 | 0 | 1 | 0 |
|  | B- (n=2) | 1 | 0 | 0 | 0 | 0 | 0 | 1 |

**Supplementary Table 02: Antibody Panel for T cell phenotyping**

| **SL*** | **Marker** | **Fluorochrome** | **Clone** | **Company** | **Catalog#** | **Dilution** |
| --- | --- | --- | --- | --- | --- | --- |
| 1 | Live/Dead | (Fixable Near-IR) | - | Thermo Fisher | L10119 | 1:1000 [Step 1] |
| 2 | CD3 | Amcyan | SK7 | BD Biosciences | 339186 | 1:100 |
| 3 | CD19 | FITC | HIB19 | BD Biosciences | 555412 | 3:100 |
| 4 | CD4 | PerCP | SK3 | BD Biosciences | 347324 | 3:100 |
| 5 | CD8 | PECy7 | SK1 | BD Biosciences | 335787 | 1:100 |
| 6 | CXCR5 | BV421 | RF8B2 | BD Biosciences | 562747 | 2:100 |
| 7 | CD45RO | PE | UCHL1 | BD Biosciences | 555493 | 3:100 |
| 8 | CD27 | APC | O323 | Thermo Fisher | 17-0279-42 | 2:100 |

*SL = Serial Number

**Supplementary Table 03: Antibody Panel for MAIT cell phenotyping**

| **SL*** | **Marker** | **Fluorochrome** | **Clone** | **Company** | **Catalog#** | **Dilution** |
| --- | --- | --- | --- | --- | --- | --- |
| 1 | Live/Dead | (Fixable Near-IR) | - | Thermo Fisher | L10119 | 1:1000 [Step 1] |
| 2 | CD3 | Amcyan | SK7 | BD Biosciences | 339186 | 1:100 |
| 3 | CD4 | PerCP | SK3 | BD Biosciences | 347324 | 3:100 |
| 4 | CD8 | PECy7 | SK1 | BD Biosciences | 335787 | 1:100 |
| 5 | TCR Vα7.2 | PE | 3C10 | Biolegend | 351706 | 2:100 |
| 6 | CD161 | APC | DX12 | BD Biosciences | 550968 | 2:100 |
| 7 | CD69 | PE-Cy5 | FN50 | BD Biosciences | 555532 | 2:100 |

*SL = Serial Number

**Supplementary Table 04: Antibody Panel for NK cell phenotyping**

| **SL*** | **Marker** | **Fluorochrome** | **Clone** | **Company** | **Catalog#** | **Dilution** |
| --- | --- | --- | --- | --- | --- | --- |
| 1 | Live/Dead | (Fixable Near-IR) | - | Thermo Fisher | L10119 | 1:1000 [Step 1] |
| 2 | CD3 | PB | SP34-2 | BD Biosciences | 558124 | 1:100 |
| 3 | CD19 | APC Cy7 | SJ25C1 | BD Biosciences | 557791 | 1:100 |
| 4 | CD14 | APC Cy7 | MφP9 | BD Biosciences | 557831 | 1:100 |
| 5 | CD16 | FITC | 3G8 | BD Biosciences | 555406 | 2:100 |
| 6 | CD56 | PerCP Cy5.5 | B159 | BD Biosciences | 560842 | 2:100 |

*SL = Serial Number

**Supplementary Table 05: Antibody Panel for AIM assay**

| **SL*** | **Marker** | **Fluorochrome** | **Clone** | **Company** | **Catalog#** | **Dilution** |
| --- | --- | --- | --- | --- | --- | --- |
| 1 | Live/Dead | (Fixable Near-IR) | - | Thermo Fisher | L10119 | 1:1000 [Step 1] |
| 2 | CD14 | BV785 | M5E2 | Biolegend | 301840 | 1:100 |
| 3 | CD16 | BV785 | 3G8 | Biolegend | 302046 | 1:100 |
| 4 | CXCR5 (CD185) | BV605 | J252D4 | Biolegend | 356930 | 1:100 |
| 5 | PD1 (CD279) | EF450 (PB) | MIH4 | ThermoFisher | 48-9969-42 | 2:100 |
| 6 | CD40L (CD154) | BV711 | 24-31 | Biolegend | 310838 | 2:100 |
| 7 | CD8 | PE-Cy7 | SK1 | BD Biosciences | 335787 | 1:100 |
| 8 | CD69 | PE-Cy5.5 | CH/4 | ThermoFisher | MHCD6918 | 2:100 |
| 9 | CD4 | Amcyan | SK3 | BD Biosciences | 339187 | 3:100 |
| 10 | CD137 | PE | 4B4-1 | Biolegend | 309804 | 1:100 |
| 11 | CD19 | FITC | HIB19 | BD Biosciences | 555412 | 1:100 |
| 12 | OX40 (CD134) | APC | Ber-ACT35 | Biolegend | 350008 | 1:100 |

*SL = Serial Number
